# The effectiveness of specialized nursing interventions for patients with Parkinson disease: A randomized controlled study protocol

**DOI:** 10.1097/MD.0000000000023972

**Published:** 2021-01-15

**Authors:** Yi Chen, Ting Lu, Xiaoyan Jiang, Xinyue Huang

**Affiliations:** a Department of Neurology; b Department of Cardiology, Sichuan Academy of Medical Sciences & Sichuan Provincial People's Hospital, Sichuan, China.

**Keywords:** Parkinson disease, protocol, specialized nursing interventions

## Abstract

**Background:**

The purpose of this experiment is to evaluate the impact of the care of Parkinson disease nurse specialist on improving motor symptoms and life quality for patients with Parkinson disease (PD).

**Method:**

This is a randomized controlled research, and it will be conducted from April 2021 to October 2021 at Sichuan Provincial People's Hospital. The experiment was granted through the Research Ethics Committee of Sichuan Provincial People's Hospital (004510293). All the patients suffer from PD, age 18 years or older, both female and male, regardless of the duration or severity of this disease are eligible. The exclusion criteria contains: lack sufficient knowledge to complete questionnaires, serious physical comorbidities or refuse to take part in the program. In our experiment, the major result measures are motor symptoms and life quality. For the measurement of life quality, we will utilize Parkinson disease Questionnaire-39, the most extensively utilized the scale of life quality in PD. The evaluation of motor symptoms severity is carried out with the revision of Unified Parkinson Disease Rating Scale sponsored by Movement Disorder Society.

**Results:**

Table 1 indicates clinical outcomes at different time points.

**Conclusion:**

The Parkinson's disease nurse specialist care may promote the life quality in the PD patients.

**Trial registration number:**

researchregistry 6284.

## 1 Introduction

Parkinson disease (PD) is a type of progressive chronic neurodegenerative disease, and it is characterized via the apparent dopaminergic neurons early death.^[1,2]^ It is the most prevalent movement disorder, and is the second most familiar central nervous system degenerative disease.^[3]^ PD is also related to the non-motor symptoms, which may be more than a decade earlier than the motor symptoms.^[4]^ In the later stages of PD, such non-motor symptoms can become troublesome. Several researches have reported epidemiological data on PD. Nevertheless, the differences in research methods make it difficult to compare the prevalence estimates directly. It is commonly believed that the incidence of this disease is between 1 and 2/1000 among the unselected population and that 1 percent of population over 60 years of age suffer from PD.^[5,6]^ PD is rare before 50 years old, and in highest age group, the incidence rate is 4%. At present, the main treatment for PD is pharmacological therapy^[7]^; nevertheless, these symptomatic therapies have great limitations for patients with advanced disease. Many disabling characteristics occur later in this disease, involving motor complications after the long-term dopamine treatment, dopamine-resistant motor symptoms, and non-motor symptoms. Despite significant progress has been made in the surgical treatment and medical treatment of PD, there is still a lack of definitive disease-modifying treatment.

More and more studies have emphasized the important role of nursing care in patients with PD.^[8–10]^ In the area of primary health care, the enhanced collaboration between nurses and doctors could result in more comprehensive and therefore better-quality health care. In view of the special PD situation, Parkinson disease nurse specialist (PDNS) may have a significant effect in a team with multidisciplinary.^[11]^ However, PDNS care seldom been used in the care of patients with PD. Therefore, we perform this randomized controlled research protocol to evaluate the influence of the care of PDNS on improving motor symptoms and life quality for the PD patients.

## 2 Methods

The experiment will be implemented from April 2021 to October 2021 at Sichuan Provincial People's Hospital. The experiment was granted through the Research Ethics Board of Sichuan Provincial People's Hospital (004510293) and recorded in research registry (researchregistry6284).

### 2.1 Randomization

This is a randomized controlled trial. Sequence of random numbers is generated by a computer. Sequentially numbered sealed opaque envelopes are used for the concealment of random numbers. All the patients participating in this study are randomly divided into control group and intervention group, with 45 patients in each group.

### 2.2 Inclusion and exclusion criteria

All the patients suffer from PD, age 18 years or older, both female and male, regardless of the duration or severity of this disease are eligible. The exclusion criteria contains: lack sufficient knowledge to complete questionnaires, serious physical comorbidities or refuse to take part in the program.

### 2.3 PDNS intervention

Intervention of PDNS will be implemented on the basis of the guideline of “Nursing care in PD”, which was issued in 2015. This involves the following:

(1) Evaluation of the individual nursing needs of PD patients and the patients’ caregivers: At the beginning of this research, PDNS conducts the specific nursing evaluations associated with the social, psychological, physical, and medical fields.
(2) Develop a treatment plan centered on patients to support the self-management of patients and their caregivers: PDNS forms a multidisciplinary program on the basis of the individual evaluation results and in accordance with the priorities of patients and caregivers.
(3) The specific nursing intervention: the intervention measures are different in disease stages, and the specific requirements and the problems of the individual patients and the patients’ caregivers are adjusted. The guideline of “Nursing care in PD” describes the specific measures and general measures for the interventions of nursing. The general measures of interventions involve providing relevant education and information, the treatment and monitoring of disease. While the specific nursing interventions contain the psychological function, urogenital function, sexual behavior, sleep, fatigue, orthostatic hypotension, the medication compliance, the burden of caregiver, self-management, mobility, coping, and the dietary problems.
(4) The collaboration with other professionals of health care. PDNS encourages and supports the multidisciplinary collaboration among the professionals of health care on the basis of the individual treatment program. The PDNS also possesses a significant effect in referral to some other health professionals in time. While the control group patients are given conventional nursing care.

### 2.4 Outcome measures

In our experiment, the major result measures are motor symptoms and life quality. For the measurement of life quality, we will utilize Parkinson disease Questionnaire-39,^[12]^ the most extensively utilized the scale of life quality in PD. The evaluation of motor symptoms severity is carried out with the revision of Unified Parkinson Disease Rating Scale sponsored by Movement Disorder Society.^[13]^

### 2.5 Statistical analysis

By using Microsoft Excel 2013, the data are recorded, and the analysis of all the data is carried out through utilizing the software of IBM SPSS Statistics for Windows, version 20 (IBM Corp., Armonk, NY). And the data are represented via the proper characteristics, for instance, median, mean, and percentage. The categorical variables and continuous variables are respectively analyzed applying the *χ*^2^-tests and independent *t*-tests. When *P* < .05, the efficacy is viewed to be statistically significant.

## 3 Result

Table 1 indicates clinical outcomes at different time points.

**Table 1:** Clinical outcomes at different time points.

Outcomes	Study group (n = 45)	Control group (n = 45)	*P* value
Quality of life at 6 mo			
Quality of life at 12 mo			
Motor symptoms at 6 mo			
Motor symptoms at 12 mo			
Self-management 6 mo			
Self-management 12 mo			
Total medical cost			

## 4 Discussion

This present study is the first randomized controlled study to assess the influence of PDNS nursing in the PD patients. PD is a kind of neurodegenerative disease, which is characterized via the progressive development of non-motor symptom and motor symptom.^[1,14]^ Some symptoms are still not fully recognized and then treated, which has a negative influence on the patients’ life quality.^[15,16]^ In addition, failure to detect and manage the symptoms in a timely manner will enhance the risk of some avoidable complications. One promising way to overcome these deficiencies is to provide the care with high quality, which requires the proactive care provided by the PDNS.^[17]^ As the professional member of a multidisciplinary team, nurses have a key effect in promoting health, treating the complications, and adapting to limitations of disease. Nurse guides the care plan to meet the needs of the patients and their families, and directs patients to seek independence associated with their behavioral, cognitive, and physiological limitations by assessing the patients’ potential. The present guidelines suggest that every PD patient should receive the care of PDNS. Nevertheless, the scientific evidence to prove the influence of the care of PDNS is scant. Hence, our purpose is to explore the influence of the care of PDNS for the PD patients.

## 5 Conclusion

The PDNS care may promote the life quality in the PD patients.

## Author contributions

**Conceptualization:** Ting Lu.

**Data curation:** Ting Lu.

**Funding acquisition:** Xinyue Huang.

**Investigation:** Xiaoyan Jiang.

**Methodology:** Xiaoyan Jiang.

**Writing – original draft:** Yi Chen.

## Abbreviations

Abbreviations: PD = Parkinson disease, PDNS = Parkinson disease nurse specialist.
